# Contacts with Patients in Croatian Family Medicine Practices—What Has Changed from 2019 to 2025?

**DOI:** 10.3390/healthcare14111536

**Published:** 2026-06-01

**Authors:** Juraj Jug

**Affiliations:** 1 Health Center Zagreb–West, 10000 Zagreb, Croatia; juraj2304@gmail.com; 2 School of Medicine, University of Zagreb, 10000 Zagreb, Croatia

**Keywords:** administrative burden, email, family medicine, telephone, virtual consultations

## Abstract

**Highlights:**

**What are the main findings?**

- From 2019 to 2025, in everyday work in Croatian family medicine practices, total daily patient contacts increased 2.7-fold, and the number of prescriptions and referrals issued doubled. The number of live examinations declined after peaking in 2024 but remained significantly higher than in 2019.
- All practices performed email consultations with patients from 2022, which accounted for approximately 28% of all virtual consultations in 2025, especially in those who had more than 1700 patients in care. Virtual consultations accounted for 70.5% of all contacts with practices.

**What are the implications of the main findings?**

- The increase in patients’ needs and the use of virtual consultations raises concerns about quality, security, and staff burnout and should be addressed through clear guidelines, better organization, and support from superiors.
- Systematic changes are crucial in reducing the number of contacts to ensure continuity and quality of care in family medicine practices.

**Abstract:**

**Background/objectives:** Virtual consultations (VC) with family medicine practices (FMPs) are effective, efficient, and useful communication tools in meeting patients’ health needs. However, data about workload and the share of each type of consultation within FMPs are not widely available. This study aimed to determine the total number and means of all patient consultations and examinations, their share, and changes from 2019 to 2025 in the FMPs of the Health Center Zagreb–West. **Methods:** Monthly reports from 2019 to 2025 (excluding 2020) performed in 48 FMPs were included and analyzed in this retrospective longitudinal observational study. Other collected data included the total number of insured people FMP cared for, the number of insured people aged 65 and over, their share, and the age and gender of the doctors working in FMP, indicating whether they were permanent or substitute. **Results:** The total number of contacts with FMP and solely VCs increased significantly (2.7 and 2.8-fold, respectively). In 2019, half of FMPs did not use email consultations, whereas in 2022, email consultations were recorded in all analyzed FMPs. Annual email consultations grew continuously from 42 in 2019 to 1572 in 2022, and to 3669 in 2025, accounting for 1.9%, 17.4%, and 19.9% of all contacts with FMP in 2019, 2022, and 2025, respectively. The share of telephone consultations ranged from 50% to 58% of all contacts (except in 2021), and their total number increased by 2.3-fold. Although the number of examinations declined after peaking in 2024, examination rates in 2025 remained higher than those observed in 2019. FMPs with more than 1700 patients performed significantly more consultations than FMPs with fewer patients (with 27.2% difference in 2019, 12.9% in 2021, and 32.5% in 2025). FMPs with older patients in care (≥30% aged 65+) had comparable VC shares in total contacts to those with younger populations. **Conclusions:** During the period examined, the number of contacts with Croatian FMPs nearly tripled while the number of examinations halved. Email consultations accounted for a fifth of all contacts and were more pronounced in FMPs who cared for more than 1700 patients. Their increasing use requires clear guidelines, improved organization, and support for healthcare workers to ensure quality care.

## 1. Introduction

Electronic mail (email) is increasingly used in healthcare as an additional communication tool between patients and doctors, aiming to improve accessibility and continuity of care [1,2]. Email can be sent to one or multiple recipients with or without attachments (documents or pictures) and can be easily accessed on desktop computers or smartphones. Research shows that a small number of patients (especially the elderly) do not communicate with a general/family medicine practice (FMP) by email. In contrast, most other patients find this method useful and practical, most often using it to renew prescriptions, send test results, and make administrative inquiries [3]. However, continuity of care is better maintained when it involves an established patient-physician relationship [4]. There is a greater risk of arguments, unprofessional behavior, and sending inappropriate content [5]. Additionally, email consultations should not substitute for a live physical examination of the patient [1,6]. Family medicine doctors in Croatia serve as “gatekeepers”, issuing referrals for indicated examinations in secondary and tertiary healthcare institutions (patients cannot obtain care in these institutions without a referral) and overseeing all recommendations made by other doctors. Furthermore, only general practitioners/family medicine specialists can prescribe medications to patients, with some prescriptions requiring a recommendation from the appropriate hospital specialist. Consequently, FMPs in Croatia are burdened with administrative work [7]. Each contact between a patient and a member of the FMP team can occur either live (face-to-face) or virtually. Live contacts may involve consultation or physical examination, while remote contacts (by telephone or email) are defined as virtual consultations (VCs).

During the COVID-19 pandemic, VCs played a crucial role in healthcare delivery, as physical contact was limited to patients with the greatest need (such as those requiring a physical examination) [8]. During this period, FMPs conducted most consultations related to the COVID-19 symptoms, diagnostic procedures, and treatment, in addition to their other responsibilities [9,10]. However, screening examinations for the early detection of chronic diseases (including cancer) were affected [11]. A recent study showed that the number of VCs now remains above prepandemic levels, but they have not replaced live contacts, indicating a dual use of resources [12]. Nevertheless, VCs appear to be as effective as live consultations for a range of conditions [13,14].

The quality of consultations with FMP members is essential for the timely recognition of urgent and important medical conditions, problem-solving, and FMP work planning, all of which significantly affect patient health outcomes [15]. Although email consultations can increase efficiency and speed for FMPs, issues of quality, privacy, data security, and liability remain widely discussed [15,16]. Despite these concerns, almost all FMPs in Croatia use email consultations in their daily work, and the number of emails sent continues to grow each year, as does the total number of consultations with patients (including telephone and live advice) [17]. This reduces the time a doctor and nurse can devote to each patient, making it necessary to consider employing a third person in the FMP (e.g., an administrator). However, video consultations with patients are not currently conducted within FMPs in Croatia. In a study by Baković et al., final-year students of the Zagreb School of Medicine (administrators) independently completed an average of 39% of the total FMP work during summer shifts, thereby increasing examination time for individual patients and improving employee satisfaction [18].

The number of patients under the care of one FMP is associated with more contacts with that FMP. A meta-analysis by Abu Dabrh et al. found that a smaller number of patients in care was related to improved patient satisfaction, continuity of care, and health promotion. However, clinical outcomes, utilization, and costs were comparable to practices with a larger number of patients in care [19].

According to the Bulletin of the Croatian Institute of Public Health (HZJZ) on the use of health care in the family medicine sector, the number of consultations, referrals, and prescriptions issued has increased each year, but the number of examinations and health care users has not changed [20]. The report does not distinguish between email and telephone consultations. Additionally, data on workload and the proportion of each consultation type within FMPs are limited. Therefore, this research aimed to determine the total number of contacts and the means of all consultations with patients, their proportions, and changes from 2019 to 2025 in FMPs of the Health Center (HC) Zagreb–West. Other objectives were to compare the number of contacts between FMPs based on the number of persons in their care, the share of persons older than 65, and the working doctor’s status in FMP (permanent or substitute). An additional objective was to identify independent predictors of a high number of consultations with FMPs.

## 2. Materials and Methods

This retrospective longitudinal observational study analyzed all email and telephone (VCs) with patients, as well as live (in-office) consultations and patient examinations, from 2019 to 2025 (excluding 2020 due to data unavailability) at the FMP of the HC Zagreb–West. Detailed monthly reports, dating before 2023, were missing from the servers and available only in paper copies. However, data from 2020 were incomplete and were, therefore, unsuitable for analysis.

**Figure 1:**
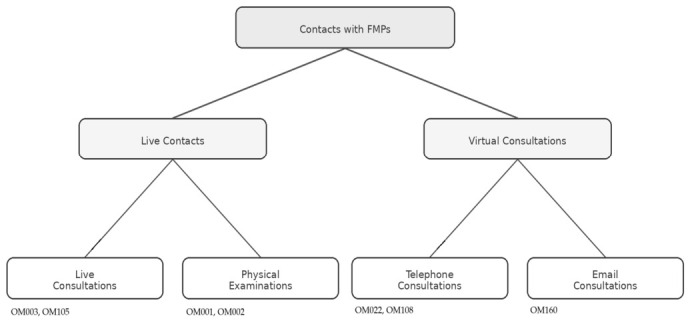
Classification of contacts with family medicine practices. Codes: OM001—first examination, OM002—follow-up examination, OM003—consultation, OM022—telephone consultation with a nurse, OM105—consultation with a patient or relative in the outpatient clinic, OM108—telephone advice to a patient or family member, OM160—e-consultation with a patient or family member.

Using available monthly practice reports obtained from the electronic system for work in each FMP, all diagnostic therapeutic procedures (DTP) with codes OM001 (first examination), OM002 (follow-up examination) and OM003 (consultation), OM022 (telephone consultation with a nurse), OM105 (consultation with a patient or relative in the outpatient clinic), OM108 (telephone advice to a patient or family member), and OM160 (e-consultation with a patient or family member) were analyzed. Procedures OM022, OM108, and OM160 were defined as VC. The classification of contacts with FMPs and corresponding DTP codes is shown in Figure 1. Each member of the FMP team (one nurse and one doctor) notes DTPs in their daily work with patients, and at the end of each month, the total numbers are listed in a monthly report for each FMP. Multiple contacts with the same patient on the same day are counted as one contact. Other data collected for each analyzed year (from 2019 to 2025) included the total number of insured people within FMP, the number of insured people over the age of 65, their share, and the age and gender of the doctor working in the practice, indicating whether they are a permanent doctor or a substitute.

At the end of 2025, the Family Medicine Department at HC Zagreb–West consisted of 71 FMPs in the urban area. Because of significant changes in FMP status, which led to its transition from private practice to HC, and vice versa, during the analyzed period, data analysis in these FMPs was not possible, and they were excluded from the research. Therefore, a total of 48 FMPs (67.6%) were included in the final analysis, caring for 78,745 patients (24,771 aged 65 years or older, 32.5%). No significant fluctuations in the number of insured people were observed from 2019 to 2025. Practices with more than 30% of patients aged 65 years or older in care were classified as FMPs with an older population, according to the estimation documented in the National Health Care Strategy document authored by the Croatian Ministry of Health [21]. To analyze the number of consultations per year, an annual period was defined as 250 working days. Based on the number of patients the practice cared for, FMPs were classified as those with ≤1700 patients (A) and those with >1700 patients (B). This threshold was chosen because the standard number of insured persons per FMP in Croatia was set at 1700 during the healthcare system reforms in the 1990s and is still in use [22].

This research has been approved by the HC Zagreb-West Ethic committee (Number: 251-12-02-21-20) and conducted in accordance with the Declaration of Helsinki.

### Statistical Analysis

Normality was assessed using the Shapiro–Wilk test, which is considered appropriate for small to moderate sample sizes. According to this test, physicians’ age and the number of patients in 2019 and 2025, including those older than 65, were distributed irregularly. Results are expressed as median [interquartile range] and percentages. The Mann–Whitney test was used to analyze the continuous variables due to their irregular distribution and small sample size. In contrast, the relationship between age and the share of insured persons aged 65 years and older, as well as the number of email consultations, was assessed using Spearman’s rank correlation. Multivariate regression analysis was performed to identify independent predictors of the number of emails, telephone consultations, live consultations, and examination changes. Variables included in multivariate models were selected based on clinical relevance and univariate associations. Assumptions of regression analysis, including multicollinearity and residual distribution, were assessed before final model construction. The analysis was performed in Microsoft Excel v.16.0. and StatSoft Statistica v.12.0.

## 3. Results

The analyzed FMPs were predominantly staffed by female physicians (44 practices, 91.7%). In 19 practices, physicians worked as substitutes (39.6%) with a median age of 28 [27–35] years. They were significantly younger than full-time physicians (49 [40–57] years, *p* < 0.001). The physicians’ younger age was moderately correlated with the total number of email consultations performed in 2022 (R = 0.384, *p* < 0.01) and 2023 (R = 0.386, *p* < 0.01). This correlation was weaker in 2024 (R = 0.280, *p* = 0.081) and 2025 (R = 0.232, *p* = 0.124). In multivariate analysis, younger physicians’ age was the only independent predictor of a higher number of email consultations in 2022 (β = −0.62, *p* < 0.001) and 2023 (β = −0.44, *p* = 0.019). On the other hand, in 2025, the only independent predictor of a higher number of email consultations was the total number of patients in FMP (β = 0.363, *p* = 0.012). The total number of patients in FMP was also the only independent predictor of a higher number of telephone consultations (β = 0.341, *p* = 0.018) and examinations (β = 0.327, *p* = 0.027), but with no association with live consultations (β = 0.200, *p* = 0.169).

### 3.1. General Changes

The total daily number of contacts with one FMP increased 2.7-fold (26.0 contacts in 2019 vs. 70.5 contacts in 2025, *p* < 0.001). A 2.3-fold increase in the median annual number of telephone consultations (4095 consultations in 2019 vs. 9371 consultations in 2025, *p* < 0.001) and emails over the years (42 consultations in 2019 vs. 3669 consultations in 2025, *p* < 0.001), as well as their share in the total number of VCs (1.0% in 2019 vs. 28.1% in 2025, *p* < 0.001), was noticed. The shares of email in all realized annual contacts with FMP from 2019 to 2025 (except 2020) were 1.9%, 10.4%, 17.4%, 18.1%, 18.7%, and 19.9%, respectively.

**Figure 2:**
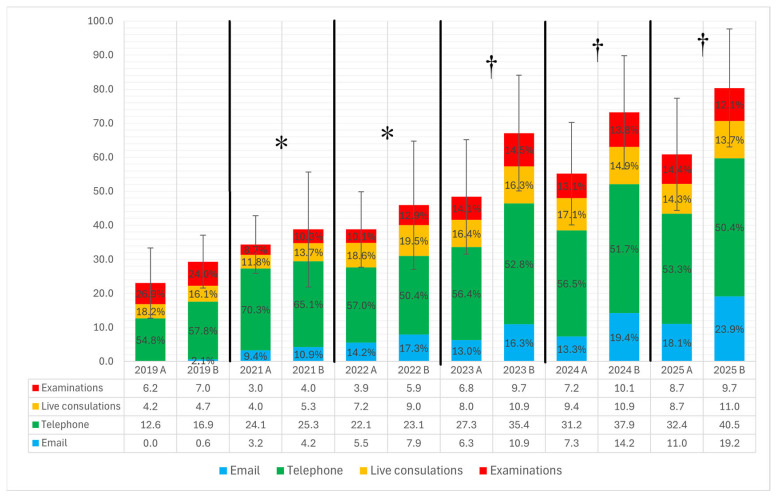
Median number (table below bars) and the share of examinations, live consultations, telephone, and email consultations per day per family medicine practice (bars) from 2019 to 2025 (except 2020), depending on the total number of persons in their care: A = ≤ 1700 patients, B = > 1700 patients. Mann–Whitney U test for differences in the total contacts with FMP between group A and B: * = *p* < 0.05, † = *p* < 0.001. The whiskers denote error bars for the total number of contacts with the practice in each year.

Combined, the number of annual VCs performed in the observed period increased 2.8-fold, from 212,538 (17.7 per day per FMP) in 2019 to 604,064 (50.3 per day per FMP) in 2025 (*p* < 0.001) (Figure 2). The total number of examinations per day was highest in 2024 (12.6 per day per FMP), 1.9-fold higher than in 2019 (6.6 per day), and then declined to 9.5 per day (24.6%) in 2025. On the other hand, the number of prescriptions issued increased from 302,925 (25.2 per day per FMP) in 2019 to 677,223 (56.4 per day per FMP) in 2025 (2.2-fold increase). A similar increase (1.9-fold) was observed in the number of issued referrals, from 139,922 (11.7 per day per FMP) in 2019 to 268,839 (22.4 per day per FMP) in 2025.

### 3.2. Differences Between Practices with Permanent and Substitute Physicians

No significant differences were found between FMPs with permanent versus those with substitute physicians (Table 1). Additionally, no significant differences were found between the two groups in the number of insured persons aged 65 years or older, nor in their share of the total number of insured persons in FMPs. The total VC ratios in all contacts with FMPs for the observed years, in chronological order, were 60.8%, 78.8%, 68.6%, 66.7%, 65.0%, and 70.5%.

### 3.3. Differences Depending on the Total Number of Patients per Practice and the Share of Patients Older than 65

Compared with 2019, when 25 FMPs (51.1%) did not conduct consultations via email, in 2022, no such practice was observed, and the share of email consultations among all consultations performed increased (Table 1). In 2025, 30 practices had more than 30% of insured people aged 65 years or older (62.5%), whereas in 2019, this share was observed in 19 practices (39.6%). In addition to the lower total number of insured persons in care (1626 [1315–1734] insured persons vs. 1767 [1707–1832] insured persons, *p* < 0.001), FMPs with an older population had equal shares of email and telephone consultations, examinations, and live advice as practices with a younger population. Compared with 2019, the total number of patients per practice overall slightly decreased (1702 vs. 1744 patients; *p* < 0.438). Still, the proportion of patients aged 65 years and older was significantly higher in 2025 (510 vs. 457 patients per FMP; *p* < 0.01).

By dividing FMPs by the number of insured persons they care for, FMPs with more than 1700 patients in care (group B, 24 FMPs, 50.0%) did 32.1% more total number of consultations in 2025 (*p* < 0.001) compared to those with fewer than 1700 patients in care (group A, 27.2% more in 2019, and 12.9% more in 2021; *p* < 0.001). During the pandemic, in 2021, smaller differences were noticed (11.5%). Throughout the entire period, no changes were noticed in the number of FMPs with more than 1700 patients. In both groups, the total number of consultations continuously increased year by year, but a significant decrease in the number and share of examinations in practices was observed in 2021 compared with 2019 (78,607 vs. 40,152 examinations in 2019; 6.6 vs. 3.3 examinations per day; *p* < 0.001). Although pandemic measures ended in 2022, the share of examinations did not increase significantly. In 2025, it is almost half of what it was before the pandemic. A significant increase in the median of telephone consultations was observed in all analyzed years (*p* < 0.001), with the most pronounced change in 2021. The share of telephone consultations in all contacts with FMPs fluctuated significantly from 2019 to 2025 (between 50.4 and 58.0%), except in 2021, when it increased significantly due to pandemic measures (70.3 and 65.1%). The share of live consultations has fallen from 17.1 to 14.3% in group A and 14.9 to 13.7% in group B over the last two years, but their median has decreased from 9.4 to 8.7 per day in group A (*p* < 0.001), but did not change in group B (from 10.9 to 11.0 per day; *p* = 0.867). The share of emails within all contacts has increased from 13.3 to 18.1% in group A, and 19.4 to 23.9% in group B, while their median has risen from 7.3 to 11.0 per day in group A (*p* < 0.001), and 14.2 to 19.2 per day (*p* < 0.001) in group B over the last two years. Median number of all daily examinations and consultations with patients in FMP for each examined year and their shares are shown in Figure 2.

**Figure 3:**
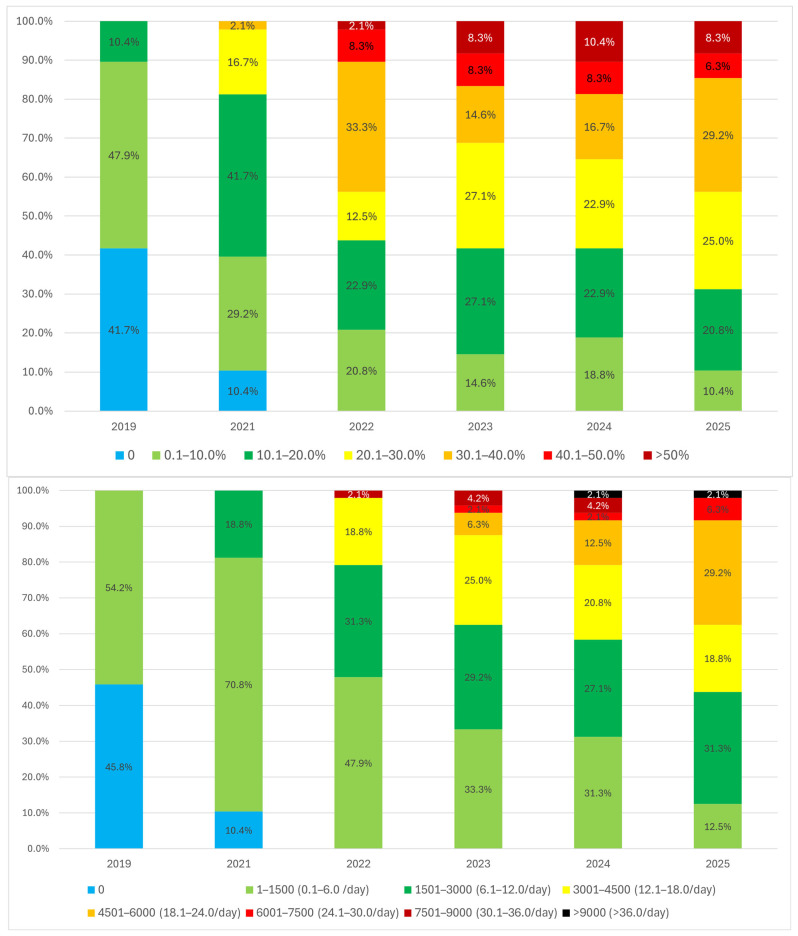
(**Above**): Share of family medicine practices (*y*-axis) by the ratio of annual email consultations among all virtual consultations with the practice, depending on the year (*x*-axis). (**Below**): Share of family medicine practices (*y*-axis) by the groups of email consultations performed annually (*x*-axis).

The upper graph in Figure 3 shows that four FMPs (8.3%) have been conducting more than 50% of their VC via email since 2023, while the number of FMPs who use email for less than 20% of their VC has decreased. On the other hand, the lower graph in the same figure shows that in 2019, half of the FMPs did not use email consultations, and among those that did, the number of emails per day was fewer than 6. The situation is markedly different in 2025, when all FMPs used email and a third of them responded to more than 18 email consultations per day.

## 4. Discussion

This research identified significant changes in both the number and nature of contacts between patients and FMPs. In 2019, email consultations were almost non-existent. Over the years, their number gradually increased, and by 2025, they accounted for slightly less than a third of all VCs with FMPs. Except in 2021, the proportion of telephone consultations remained largely unchanged, representing approximately half of total patient contacts. Similarly, Mesiano et al. found that telephone consultations increased during the COVID-19 pandemic but declined afterwards [12]. The share of VC rose significantly from 60.8% to 78.8% in 2021, then gradually declined until 2025, when it increased again to 70.5%. The total number of contacts with FMPs increased steadily from 2019 to 2025 (2.7-fold), with no significant differences between practices with a substitute and those with a permanent doctor. The number of referrals and prescriptions issued also doubled. Despite lower IT literacy among the older population, research shows that the use of email consultations in this age group can lead to better control of chronic non-communicable diseases and mental health conditions [23,24]. Given their widespread use and availability, VCs in FMP can accelerate the realization of health care rights, especially for patients who cannot physically reach a doctor or whose FMP is less accessible (e.g., in rural areas) [25]. However, the main problems highlighted in email consultations are the lack of physical contact and the inability to perform a physical examination, which means that details often important for establishing an accurate diagnosis and treatment cannot be observed [26]. Similar issues arise with telephone consultations; therefore, clinical decision-making via VC is recommended only for those patients whom a doctor has already examined in the FMP [27]. Finally, VC has the potential to transform the delivery of family medicine services by substantially reducing costs for both patients and providers in the future [28].

The differences in the amount of work performed were substantial between FMPs with more than 1700 patients and those with fewer patients. No time is reserved for VC during the FMP’s working hours, and email consultations are not yet a contractual obligation to the insurer. However, as of 2022, all FMPs conduct email consultations. Additionally, the way VC is performed, the communication style, and the IT skills of healthcare workers vary considerably across FMPs. Given that, in Croatia, the working hours of the FMP, excluding breaks and time for home visits, are six hours, it is possible that the increase in VCs may have influenced the organization and delivery of patient management within FMPs [29]. There is currently no consensus in the profession regarding which procedures should be conducted via email or telephone consultations, and which require live consultation or examination (these decisions depend on the subjective judgement of healthcare professionals). The approach to patients via VC does not differ significantly from live consultations. However, such consultations are shorter and may affect the patient’s perception of the physician. Due to the growing health needs of patients, the increasingly demanding administrative requirements, and the proliferation of new drugs and treatment modalities, VCs are crucial to the effective organization and management of the large volume of work in the FMP [30,31,32]. Conversely, prevention is a fundamental aspect of FMPs’ work, and cannot be adequately discussed with patients via email [33]. Additionally, nonverbal cues expressed online may lose some of their impact compared to those cues in offline contexts [34]. Finally, for a small number of patients, email consultations provide an additional channel to express their anxiety and concerns. These patients often overuse email consultations with lengthy and complex, sometimes nonmedical or redundant messages, which has become the most common concern among FMPs [35].

This research also found substantial variation in the number of email consultations conducted by individual practices (e.g., 12.5% of FMPs responded to fewer than six emails per day, whereas 2.1% responded to more than 36 emails per day). This example illustrates the impact of the organization of individual FMPs on patient consultations. Given the volume of VC that is performed daily, it is necessary to clearly define the work that can be conducted in this way, educate patients about the limitations of VC, ensure secure information transfer channels (such as secure domains, applications with two-factor authentication, etc.), and systematize the working hours of the FMP [28].

As early as 2017, research by Fisher et al., based on interviews with 34 doctors in the United Kingdom, highlighted the need to increase patient responsibility for their own health and behavior within the healthcare system, stating that doctors should not be responsible for educating patients about these matters. It also emphasizes the need for administrators (scribes) and the division of work among members of the FMP [18,36]. Healthcare workers in the FMPs are described as resourceful and adaptable to new demands and challenges within the healthcare system.

Healthcare workers often equate administration with paperwork, but coordination of care (such as appointment planning at FMP or hospital, consulting colleagues to resolve professional or insurance dilemmas, etc.) is also a very important skill that requires time and cannot be measured by DTPs [37]. Poor health literacy in the general population can also increase the workload in FMPs [38]. Finally, the work of the FMP could be divided among its closest associates (pharmacists, social workers, administrators working at insurers, etc.) and/or facilitated with new IT solutions [36]. The observed increase in workload and VCs may raise concerns about professional workload and healthcare quality; however, these outcomes were not directly evaluated and should be examined in future studies.

Accordingly, future studies should explore the relationship among the number of daily patient contacts, the age structure of patients in care, the number of medical errors, and the burnout among medical workers in FMPs. These results could encourage systematic changes within the healthcare system and primary care to manage the increase in contacts with FMPs efficiently.

### 4.1. Strengths

This research was conducted over six years within the same FMPs, using the same registered and performed DTPs throughout. In this way, data is collected more precisely, providing a valuable insight into VC changes in FMPs during and after the COVID-19 pandemic.

### 4.2. Limitations

A small proportion of performed contacts is likely not recorded. DTP data aggregation may also vary among FMPs due to their location, the age, and IT literacy of their members. Multiple daily consultations (such as two or more emails or phone calls) with patients are recorded as a single DTP in the system. DTP data quality validation is not possible. Although the change in the number of insured people per FMP was minimal, the population that FMPs cared for was significantly older in 2025. This study analyzed only FMPs in Zagreb (the capital of Croatia) and cannot be generalized to the entire country. Approximately one-third of the initially identified practices were excluded due to administrative changes and incomplete data availability. This may have introduced selection bias and could limit the generalizability of the findings. Because detailed comparative data from excluded practices were unavailable, the results should be interpreted with caution.

The absence of data from 2020 is a significant limitation because this period coincided with the initial implementation of pandemic-related organizational changes and the rapid expansion of VCs. Therefore, temporal trends between 2019 and 2021 should also be interpreted with caution. Because repeated measurements from the same practices were analyzed over multiple years, observations may not have been fully independent. More advanced longitudinal statistical methods, such as mixed-effects models or generalized estimating equations, could provide more robust estimates of temporal trends.

Overall, differences in documentation practices between FMPs, potential underreporting of consultations, and the lack of longitudinal statistical adjustment may have influenced the observed results. Additionally, the study did not assess clinical outcomes, patient satisfaction, or healthcare quality indicators, which limits the interpretation of the broader impact of increasing VCs.

## 5. Conclusions

This study found a significant 2.7-fold increase in the total number of annual contacts between patients and Croatian FMPs from 2019 to 2025. Care delivery shifted towards remote modalities (70.5% of all contacts, 19.9% via email) with marked between-practice variability. VCs did not replace live examinations. In fact, the number of live examinations declined after peaking in 2024 but remained significantly higher than in 2019. These changes were more pronounced in FMPs caring for more than 1700 patients, with no significant differences between FMPs with a higher proportion of patients over 65 or those with a permanent or substitute physician. The total number of prescriptions and referral papers issued doubled in just 6 years. As VCs will remain an integral part of FMPs’ work, it is essential to clearly define their role and structure, allocate sufficient time for their performance within working hours, and further investigate their impact on healthcare quality.

## Figures and Tables

**Table 1:** Differences in demographic parameters and the median annual number of consultations from 2019 to 2025 between family medicine practices (FMP) with a permanent physician working and with a substitute physician.

		All *N* = 48 (Median [IQR])	FMP with:	
			A Permanent Physician, *N* = 29 (Median [IQR]) †	A Substitute Physician, *N* = 19 (Median [IQR]) †
**Physician’s age (years)**		40 [28–54]	49 [40–57] *	28 [27–35] *
**Number of patients per FMP (2019)**		1744 [1505–1905]	1670 [1500–1905]	1751 [1522–1852]
**Number of patients older than 65 years (2019)**		457 [384–529]	463 [398–541]	455 [401–527]
**Share of patients older than 65 years (%, 2019)**		26.9 [24.2–33.8]	27.1 [24.1–34.7]	26.3 [23.4–33.5]
**Number of patients per FMP (2025)**		1702 [1447–1776]	1720 [1477–1780]	1686 [1377–1752]
**Older than 65 years (2025)**		510 [432–622]	512 [435–601]	502 [432–624]
**Total number of email consultations**	**2019**	42 [0–246]	54 [0–192]	24 [0.0–324]
	**2021**	972 [454–1270]	1008 [375–1266]	870 [456–1440]
	**2022**	1572 [846–2697]	1506 [750–2592]	1662 [1218–2718]
	**2023**	2245 [1272–3905]	2121 [967–3903]	2708 [1357–3940]
	**2024**	2661 [1301–4277]	2866 [1117–4104]	2531 [1307–5040]
	**2025**	3669 [2135–5011]	3711 [2132–4916]	3002 [2247–5039]
**Total number of telephone consultations**	**2019**	4095 [3006–5145]	3614 [2940–4548]	4266 [3090–5670]
	**2021**	6201 [5257–8016]	5904 [5283–7974]	6588 [5214–8058]
	**2022**	5642 [4309–6846]	5484 [4082–6090]	6345 [4734–7122]
	**2023**	7515 [4863–9375]	5973 [4383–9272]	8531 [6822–9592]
	**2024**	8536 [6355–10,429]	7470 [5149–9891]	9340 [7329–10,532]
	**2025**	9371 [7177–10,944]	8422 [6967–10,939]	9816 [8111–11,128]
**Share of email consultations in the total number of virtual consultations (%)**	**2019**	1.0 [0.0–5.5]	1.5 [0.0–5.2]	0.6 [0.0–7.1]
	**2021**	13.9 [5.8–17.6]	14.6 [5.6–17.3]	11.7 [6.2–20.2]
	**2022**	21.8 [14.1–32.5]	21.5 [13.3–32.8]	20.8 [15.8–32.2]
	**2023**	23.0 [15.1–37.0]	26.2 [12.7–38.8]	24.1 [16.2–32.3]
	**2024**	23.8 [12.2–38.5]	27.8 [10.1–35.0]	21.3 [15.0–39.0]
	**2025**	28.1 [17.7–36.5]	30.9 [15.5–38.0]	23.4 [19.4–32.3]
**Total number of examinations**	**2019**	1637 [1266–1998]	1590 [1278–1852]	1950 [1206–2208]
	**2021**	918 [474–1113]	756 [462–1026]	978 [532–1146]
	**2022**	1185 [717–1746]	1182 [684–1728]	1296 [732–1794]
	**2023**	2181 [1505–2614]	2332 [1830–2760]	1876 [1320–2347]
	**2024**	2183 [1656–2872]	2139 [1931–2625]	2227 [1518–2888]
	**2025**	2284 [1944–2745]	2260 [1931–2770]	2380 [1957–2720]

Mann–Whitney U test between family medicine practices (FMPs) with the same doctor working and with a substitute was used. *—significant differences at *p* < 0.05. †—unadjusted for patient structure and physicians’ age. IQR = interquartile range.

## Data Availability

This data indirectly presents the realized income and working methods of individual family medicine practices within the Health Center Zagreb West. This data is not classified, but the Health Center needs to know who accessed it and why. I can provide this data for your archive if necessary, but it should not be publicly available without any control.

## Abbreviations

The following abbreviations are used in this manuscript:

FMP	Family medicine practice
HZJZ	Croatian Institute of Public Health
VC	Virtual consultation
HC	Health Center
DTP	Diagnostic therapeutic procedure
FTF	Face-to-face
